# Investigating the impact of written emotion disclosure on the level of occupational stress among intensive care nurses

**DOI:** 10.3389/fpsyg.2022.1064189

**Published:** 2023-03-01

**Authors:** Zahra Jafariathar, Shayesteh Haghighi, Simin Jahani, Elham Maraghi

**Affiliations:** ^1^Department of Nursing and Midwifery, Nursing Care Research Center in Chronic Disease, Ahvaz Jundishapur University of Medical Sciences, Ahvaz, Iran; ^2^Medical and Surgical Nursing Department, Nursing and Midwifery School, Nursing Care Research Center in Chronic Diseases, Ahvaz Jundishapur University of Medical Sciences, Ahvaz, Iran; ^3^Department of Nursing and Midwifery, Nursing Care Research Center in Chronic Disease, Ahvaz Jundishapur University of Medical Sciences, Ahvaz, Iran; ^4^Department of Biostatistics and Epidemiology, Faculty of Public Health, Ahvaz Jundishapur University of Medical Sciences, Ahvaz, Iran

**Keywords:** occupational stress, nurses, rewriting, intensive care unit, emotion

## Abstract

**Objective:**

The present study was conducted with the aim of determining the impact of rewriting pleasant events on the level of occupational stress in Intensive Care Unit (ICU) nurses.

**Methods:**

This is a quasi-experimental research conducted on nurses working in the intensive care units of Imam Khomeini and Golestan hospitals in Ahvaz, from July to November 2021. Seventy-six nurses were selected based on the inclusion criteria, and were then randomly assigned to the intervention and the control groups. The nurses’ demographic information form and the Expanded Nursing Stress Scale (ENSS) were used to collect data. In the intervention group, the technique of rewriting pleasant events was used for 8 weeks, at least once a week. The data was analyzed with SPSS V20.

**Results:**

No significant difference in the demographic characteristics was observed between the intervention and the control groups (*p* > 0.05). The pre-intervention occupational stress of the nurses in the intervention and the control groups were reported to be 173.86 ± 26.75 and 173.05 ± 24.47, respectively, showing no statistically significant difference (*p* = 0.89). After the intervention, the occupational stress scores were 134.21 ± 16.09 and 172.36 ± 24.33, respectively, showing a significant difference between the two groups (*p* < 0.001).

**Conclusion:**

Considering the impact of rewriting pleasant events on the level of occupational stress of ICU nurses, it is recommended that nursing managers and nurses plan training programs for nurses in this area, and encourage other nurses to do so, too. It is also suggested to implement this intervention on the nurses of other wards as well.

## Introduction

One of the most important stressors in every one’s life is his/her job, and today, occupational stress has become a common and costly problem in the workplace (Alkhawaldeh et al., 2020). Occupational stress can be regarded as the accumulation of stressful factors and job-related conditions, upon the stressfulness of which most people agree (Hazavehei et al., 2017). Among different occupational groups, healthcare workers, especially those working in the hospital environment, experience higher occupational stress (Ribeiro et al., 2018), and among health care jobs, nursing is known for its high risk of fatigue and disease (Vahedian-Azimi et al., 2019).

The National Institute for Occupational Safety and Health of the United States has placed nursing at the top of the 40 professions with a high prevalence of stress-related diseases (Hazavehei et al., 2017).

The results of various studies show that job stress leads to leaving the job, conflicts among colleagues, health disorders, job dissatisfaction (Bonzini et al., 2015), reduced creativity, and job results decrease in the ability to make correct and timely decisions, feelings of incompetence and depression, disgust and fatigue from work, decrease in energy and work efficiency, and decrease in the quality of nursing care. Also, people with high work stress are more likely to have work accidents (Moayed et al., 2015). Despite the fact that nursing itself is stressful, in some wards, this stress is even multiplied (Torkaman et al., 2019). Close observation of health care conflicts, patients’ pain and suffering, delays in clinical decision-making for end-of-life patients, and the inappropriate use of medical resources expose ICU nurses to the highest level of occupational stress (Torkaman et al., 2019). Nouroozi Kushali et al. (2013) reported ICU nurses’ levels of stress, anxiety and depression as 33, 33.9, and 30.8%, respectively. Torkaman et al. (2019) describe this intensity from moderate to severe. If this stress is ignored, it will cause many problems for nurses, such as sleep disorders, digestive disorders, general health decline, decreased functioning, reduced quality of care, job burnout, and leaving the job (Poursadeghiyan et al., 2017).

In addition to the mentioned problems, today, with the spread of the epidemic of COVID-19 (Corona Virus Disease), a high point has been created in the aggravation of the mental problems of the medical staff, especially nurses, due to direct contact with the patients of COVID-19 (Fernandez et al., 2020; Zhang et al., 2021).

Previous researches have reported that during the outbreak of infectious diseases such as influenza and Ebola, a wide range of psychosocial effects were created on people at the individual, social and international levels, which at the individual level due to the high rate of death caused by these viruses, Psychological symptoms and problems, including job stress, increased (Boshra et al., 2020; Fernandez et al., 2020).

In order to reduce occupational stress, several actions and interventions have been recommended, including the use of food supplements such as zinc supplements (Baradari et al., 2013), educational programs and interventions based on appropriate theories and models of health education (Hazavehei et al., 2017), interventions based on emotion regulation (Hatamian, 2020), encouraging nurses to actively participate in clinical decision-making, developing supportive systems, providing opportunities for professional growth (Dolatshad et al., 2020), and creating a spirit of cooperation between physicians and nurses instead of authoritative and hierarchical relationships (Lloyd and Campion, 2017). Using psychological interventions focusing on emotion is also a useful approach, a simple way in which emotions are expressed through sharing and writing pleasant memories (Fadaei et al., 2020). Studies show that psychological debriefing is a proper method for preventing anxiety and mood disorders after accidents (Muosavi et al., 2013). In this regard, the results of the study by Keene et al., (2010) showed that debriefing sessions can be an effective approach to support health care providers in managing the grief caused by the death of a child. An appropriate approach toward implementing these potential interventions is written emotion disclosure (WED). This approach is a type of writing therapy, first introduced by Pennebaker and Beall in 1986. WED typically includes the participants who write down their experiences of a traumatic incident for 3–5 days later, 15 to 30 min a day (Riddle et al., 2016). In recent years, studies have been conducted to investigate the effects of expressing positive life experiences. The initial findings show that expressing positive and pleasant experiences can yield many benefits such as life satisfaction, and reducing health complaints (Wing et al., 2006; Burton and King, 2008). For example, Folkman argued that positive emotions create psychological *respite* from stressors and the related negative emotions (Folkman, 1997). Fredrikson’s Broaden and Building Theory suggests that positive emotions expand people’s thought-action repertoires, thus helping override thoughts and actions associated with negative stress-induced emotions such as depression and anxiety.

In this regard, a study was conducted by Mirzazadeh et al. (2015) which showed that holding debriefing sessions for describing stressful events can have a positive impact on nurses’ moral distress. However, some studies reported different results, such as the study by Ebadi et al. (2021), where it was found emotion disclosure through writing increases the stress among the mothers of the children with autism. It should be noted that other studies had different target populations such as the mothers of autistic children, and the type of events had been different as well; Mirzazadeh’s study investigated the expression of stressful incidents. On the other hand, there exists a limited number of similar studies, and further research is needed to develop approaches for reducing occupational stress among ICU nurses. Therefore, due to the importance of the quality of their performance, this research aims to evaluate the impact of written emotion disclosure on the occupational stress of ICU nurses. The hypothesis of this study is writing emotion disclosure reduces occupational stress in nurses working in intensive care units.

## Materials and methods

This is a quasi-experimental intervention study whose population consists of nurses working in the ICUs and the Critical Care Units (CCUs) of Imam Khomeini and Ahvaz Golestan hospitals, from July to early November 2021. The samples size was calculated to be 38 subjects per group, based on the data obtained from the previous studies (Saedpanah et al., 2016), and *α* = 0.05, power = 80% (*β* = 0.8), *d* = 20.4, *s* = 28.5, and taking into account a 15% attrition rate. The samples were selected based on the inclusion criteria such as working experience in ICU and CCU for at least 1 year, and holding at least a bachelor’s degree in nursing. The exclusion criteria consisted of withdrawing from the research and the subjects’ changing their workplace to another department.

The data collection tool consisted of a form for nurses’ demographic data (age, gender, the level of education, clinical work experience, the type of employment, and working shifts), and the Expanded Nursing Stress Scale (ENSS). The ENSS was designed and validated by Gray-Toft and Anderson (1981) and includes 57 items on a 5-point Likert scale. This questionnaire examines the level of occupational stress in the research units.

There are 9 dimensions in this tool including death and dying (7 items), conflict with physicians (5 items), inadequate emotional preparation (3 items), peer-related problems (6 items), supervisor-related problems (7 items), workload (9 items), treatment uncertainty (9 items), patients and their families (8 items), and discrimination (3 items). The items are answered as I am not stressed at all (Alkhawaldeh et al., 2020), I am sometimes stressed (Hazavehei et al., 2017), I am stressed most of the time (Ribeiro et al., 2018), I am extremely stressed (Vahedian-Azimi et al., 2019), and this situation does not apply to my duties (Bonzini et al., 2015). If someone has not ever faced such a situation, he/she chooses 0. The scores range from 57 to 285, where a score of 57 to 114 indicates a low level of occupational stress among the target population, and a score between 114 and 228, a moderate occupational stress. A score above 228 shows a high level of occupational stress (Ghanei Gheshlagh et al., 2013; Shareinia et al., 2018). The ENSS is an international credible questionnaire whose validity and reliability has been examined many times in different parts of the world In Iran, the instrument was validated by Ghanei Gheshlagh et al. (2013). The Cronbach’s alpha coefficient for the subscales ranged from *α* = 0.65 (discrimination) to *α* = 0.88 (conflict with nurses) (Shareinia et al., 2018).

After obtaining permission from the ethics committee and research assistant of Jundishapur University of Medical Sciences, Ahvaz, the researcher went to the research environment including ICUs and CCUs of Imam Khomeini and Golestan hospitals in Ahvaz and after introducing herself to the officials and presenting the letter of introduction and fully and accurately explaining the objectives of the research to them, she obtained the approval of the officials to conduct the research.

Then, the list of nurses who met the entry criteria was obtained from the head of the unit, and after introducing themselves and explaining the objectives of the research to the nurses, written informed consent was obtained from the eligible people willing to participate in the study.

It should be mentioned that in order to attract the attention of nurses, full explanations were provided regarding the way of conducting the study.

Then, the selected samples were allocated to intervention and control groups based on block classification. The people of the intervention group were divided into groups of 6 people and were trained in the conference room of the hospital departments.

At the beginning, the level of occupational stress in both intervention and control groups was measured using the ENSS. Then a short two-hour session was held to introduce research objectives and methods to the intervention group participants and teach them how to fill in the notebook. For 8 weeks, at least once a week, all the participants in the intervention group were asked to write down their thoughts and feelings about pleasant topics and positive events they had experienced at work in the provided notebooks, including the best working experiences, the best behaviors regarding patients, colleagues, doctors, supervisors, patients’ relatives, and the service staff, regardless of the limitations of writing style, sentence structure or grammar (Ashley et al., 2011). They were also asked to try to write their notes in a peaceful place, while being alone, and then record the date and the time for each note, and give it to the researcher at the end. The notebook which was provided to the intervention group subjects contained 20 A5 papers. The first 4 pages of the notebook included the research objectives and method, a description of occupational stress, rewriting the events, and an example of these writings. The rest 16 blank pages were for writing the thoughts, feelings, and events experienced by the subjects. The intervention lasted for 8 weeks. At the end of the study, the ENSS was again filled out by both groups. In order to comply with ethical principles, at the end of the study, a workshop was held for the control group participants to teach them how to record their positive thoughts and feelings as well as their work experiences.

Data analysis was done using SPSS V20. The quantitative variables were reported using mean and standard deviation, and qualitative variables, using frequency (percentage). The normality of the distribution of quantitative variables was determined using the Shapiro–Wilk test. The comparison of qualitative variables was independently performed in the two groups, using Fisher’s exact test and chi-square test. The quantitative variables in the two groups were compared using the paired *t*-test or its non-parametric equivalent (Mann–Whitney test). Univariate effects of intervention condition on posttest outcome measures were examined using between-subjects analysis of covariance (ANCOVA), adjusting for pretest scores.

The significance level of the tests was considered as 0.05. In order to comply with ethical principles, the research was approved by the Ethics committee of Ahvaz Jundishapur University under the code IR.AJUMS.REC.1400.154, and the necessary permits were obtained. The researcher also introduced the research team and the research objectives to the participants, and reminded them that participation was absolutely voluntarily, with no impact on their evaluation process, and that the subjects’ data would remain confidential. It should be noted that because this study was conducted on nurses, it was not considered as a clinical trial and therefore did not need to receive the code “irct.ir.”

## Findings

**Figure 1:**
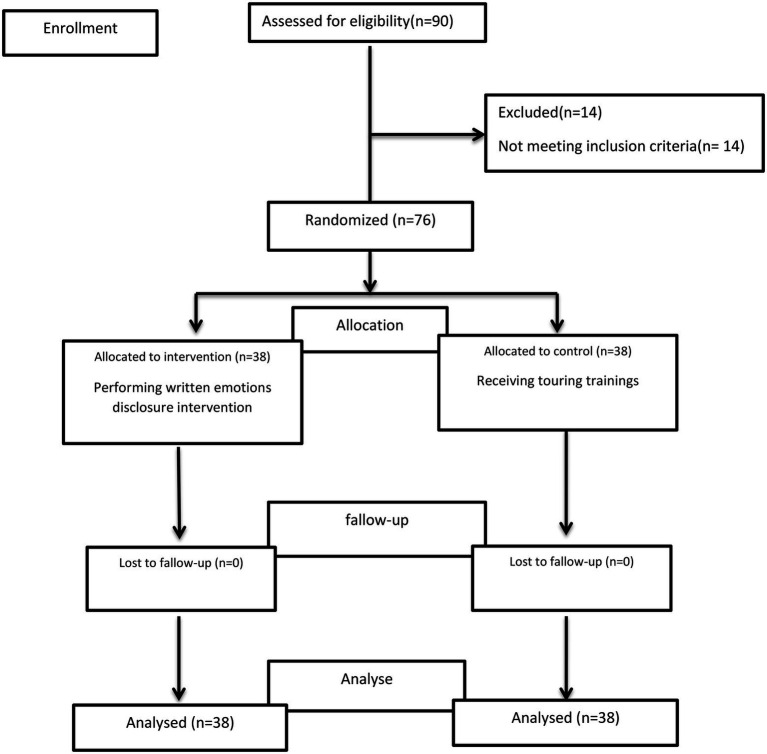
Flow diagram for participants included in study.

Seventy-six nurses participated in this study, and none withdraw from the study or were excluded (Figure 1). Based on the results, no significant differences in gender (*p* = 0.77), marital status (*p* = 0.64) and the level of education (*p* = 0.51) were observed between the intervention and the control groups according to Fisher’s exact test. No significant differences in employment status (*p* = 0.81) and work shift (*p* = 0.35) were observed between the groups according to chi-square test, and none was observed in nurses’ age (*p* = 0.74) and working experience (*p* = 0.73) according to Mann–Whitney test, either (Table 1).

**Table 1:** Comparison of the absolute frequency and percentage of relative frequency of nurses according to demographic variables.

Variable	Categories	Intervention	Control	*p*-value
		*N* (%)	*N* (%)	
Sex	Male	9 (23.7)	7 (18.4)	0.77*
	Female	29 (76.3)	31 (81.6)	
Marital status	Single	17 (44.7)	14 (36.8)	0.64*
	Married	21 (55.3)	24 (63.2)	
Education level	BSN	31 (81.6)	34 (89.5)	0.51*
	MSc	7 (18.4)	4 (10.5)	
Employment status of nurses	Official nurse	27 (71.1)	24 (63.2)	0.81**
	Contractual nurse	5 (13.2)	5 (13.2)	
	Newly graduated nurse	2 (5.3)	4 (10.5)	
	Unemployed nurse	4 (10.5)	5 (13.2)	
Shift work	Morning	7 (18.4)	3 (7.9)	0.35**
	Evening	3 (7.9)	8 (21.1)	
	Night	2 (5.3)	1 (2.6)	
	Long day	6 (15.8)	3 (7.9)	
	Morning - evening	2 (5.3)	3 (7.9)	
	Rotation	18 (47.4)	20 (52.6)	
		*M* ± SD	*M* ± SD	
Age		35.81 ± 4.72	35.21 ± 5.09	0.74***
work experience		12.68 ± 4.24	12.52 ± 4.70	0.73***
work experience in ICU		5.92 ± 3.78	6.76 ± 4.20	0.57***
Number of night shifts		2.84 ± 2.49	3.21 ± 2.18	0.35***

^*^Fisher’s exact test, ^**^Chi-square test, ^***^Independent *t*-test.

According to Table 2, the mean and the standard deviation of the total pre-intervention scores of nurses’ occupational stress were 173.86 ± 26.75 and 173.05 ± 24.47 in the intervention and the control group, respectively, showing no statistically significant difference based on independent *t*-test (*p* = 0.89). After the intervention, the mean and the standard deviation of the total occupational stress scores reached 134.21 ± 16.09 in the intervention group, and 172.36 ± 24.33 in the control group, indicating a significant difference according to the independent *t*-test (*p* < 0.001). In addition, prior to the intervention, no significant difference between the dimensions was seen in both groups. However, after the intervention, in all the dimensions, except the dimension *conflict with physicians* (*p* = 0.93) the stress score decreased significantly in the intervention group (*p* < 0.05).

**Table 2:** Comparison of the average scores of total occupational stress and its dimensions in nurses before and after the intervention in two groups.

Variable	Time	Intervention	Control	*p*-value
		*M* ± SD	*M* ± SD	
Occupational stress	Pre-test	173.86 ± 26.75	173.05 ± 24.47	0.89
	Post-test	134.21 ± 16.09	172.36 ± 24.33	0.001
*p*-value		0.001	0.26	
Death and dying	Pre-test	21.57 ± 4.18	22.47 ± 4.01	0.34
	Post-test	17.02 ± 3.75	22.36 ± 3.19	0.001
*p*-value		0.001	0.16	
Conflict with physicians	Pre-test	13.00 ± 1.93	12.44 ± 3.08	0.35
	Post-test	12.26 ± 2.46	12.31 ± 3.11	0.93
*p*-value		0.056	0.16	
Inadequate emotional perception	Pre-test	10.18 ± 3.01	11.21 ± 2.70	0.12
	Post-test	6.39 ± 2.18	11.15 ± 2.68	0.001
*p*-value		0.001	0.32	
Problems with peers	Pre-test	18.07 ± 2.85	11.18 ± 1.82	0.10
	Post-test	12.10 ± 2.36	16.94 ± 1.64	0.001
*p*-value		0.001	0.095	
Problems with supervisors	Pre-test	19.84 ± 2.64	18.76 ± 3.61	0.14
	Post-test	16.65 ± 2.93	18.63 ± 3.79	0.013
*p*-value		0.001	0.65	
Workload	Pre-test	26.44 ± 5.44	25.10 ± 4.83	0.24
	Post-test	22.76 ± 3.92	25.28 ± 4.59	0.012
*p*-value		0.001	0.07	
Uncertainty concerning treatment	Pre-test	32.21 ± 7.50	33.68 ± 7.25	0.38
	Post-test	22.21 ± 4.62	33.76 ± 7.71	0.001
*p*-value		0.001	0.89	
Patients and family	Pre-test	22.39 ± 3.19	21.18 ± 3.54	0.12
	Post-test	19.34 ± 3.29	20.97 ± 3.68	0.04
*p*-value		0.001	0.14	
Discrimination	Pre-test	10.13 ± 2.86	11.00 ± 2.95	0.19
	Post-test	5.44 ± 1.98	10.92 ± 3.01	0.001
*p*-value		0.001	0.32	

There was a statistically significant effect between two groups according to posttest outcome measures adjusting for pretest scores for all dimensions except for conflict with physician’s dimension (Table 3).

**Table 3:** Descriptive statistics and ANCOVA results for the outcome measures.

Outcome	Intervention	Control	*F*	*p*	Partial Eta-square
Death and dying			76.07	<0.0001	0.51
Baseline	21.57 ± 4.18	22.47 ± 4.01			
After intervention	17.02 ± 3.75	22.36 ± 3.91			
Conflict with physicians			1.99	0.162	0.02
Baseline	13.00 ± 1.93	12.44 ± 3.08			
After intervention	12.26 ± 2.46	12.31 ± 3.11			
Inadequate emotional preparation			109.52	<0.0001	0.6
Baseline	10.18 ± 3.01	11.21 ± 2.70			
After intervention	6.39 ± 2.18	11.15 ± 2.68			
Peer-related problems			184.79	<0.0001	0.71
Baseline	18.07 ± 2.85	17.18 ± 1.82			
After intervention	12.10 ± 2.36	16.94 ± 1.64			
Supervisor-related problems			24.05	<0.0001	0.24
Baseline	19.84 ± 2.64	18.76 ± 3.61			
After intervention	16.65 ± 2.93	18.63 ± 3.79			
Workload			47.87	<0.0001	0.39
Baseline	26.44 ± 5.04	25.10 ± 4.83			
After intervention	22.76 ± 3.92	25.28 ± 4.59			
Treatment uncertainty			144.1	<0.0001	0.66
Baseline	32.21 ± 7.50	33.68 ± 7.25			
After intervention	22.21 ± 4.62	33.76 ± 7.71			
Patients and their families			33.8	<0.0001	0.31
Baseline	22.39 ± 3.19	21.18 ± 3.54			
After intervention	19.34 ± 3.29	20.97 ± 3.68			
Discrimination			140.34	<0.0001	0.65
Baseline	10.13 ± 2.86	11.00 ± 2.95			
After intervention	5.44 ± 1.98	10.92 ± 3.01			

## Discussion

The present study was conducted with the aim of determining the effect of written emotion disclosure on the level of occupational stress in nurses working in intensive care units. According to the results of the study, the mean and the standard deviation of pre-intervention occupational stress scores were not significantly different in the two groups. However, after the intervention, this difference was significant, showing a significant decrease in the intervention group.

Various studies have reported that learning nursing ethics through a written narrative tailored to nurses’ feelings helps to revive their thoughts and attitudes toward patients, and writing a narrative in nursing ethics education can lead to ethical performance (Tsuruwaka and Asahara, 2018).

Also, other studies have shown that both oral and written approaches are effective in reducing the care burden, stress and anxiety of family and youth caregivers and reducing the symptoms of depression, anxiety and stress of war wounded veterans who suffered from post-traumatic stress disorder. Meanwhile, the written approach of disclosing feelings has been more effective in reducing the burden of care and anxiety (Niles et al., 2013; Monazamitabar et al., 2015; Harvey et al., 2018).

The results of the studies by Ebadi et al. (2021) and Ashley et al. (2011) also showed that the disclosure of written emotions caused an increase in stress in caregivers or did not have an effect on reducing the psychological distress of informal caregivers, which is not in line with the present study. This difference can be interpreted in such a way that people deeply understand their problems by writing down their feelings, and with the disappearance of mental distractions, they are more affected and thus experience higher levels of stress (Ashley et al., 2011; Ebadi et al., 2021).

Also, the results of other studies showed that narrative writing had no effect on reducing the intensity and frequency of moral discomfort in intensive care nurses (Saidi et al.’s study), which according to the researcher, several factors can cause the different and inconsistent results of these studies compared to The present research will explain. The most important factors are: different target populations, time and place of research, research tools, number of samples, dependent variable and duration of briefing sessions (Saeedi et al., 2019).

Another result of the study was that after the intervention, the scores of all the dimensions of occupational stress, except conflict with physicians, decreased in the intervention group. The effectiveness of the written disclosure method in the overall scores of the occupational stress among intensive care unit nurses and most of its dimensions is an important finding, showing that this method can be used to reduce various aspects of stress. In regard with conflict with physicians, it seems that disclosing feelings does not reduce conflict with the physicians, the reasons of which are also different. Since this conflict is caused by reasons such as physicians’ looking down on other medical workers, receiving organizational support, delegating their own duties to nurses, not fully implementing rules and regulations, and nurses and physicians’ having misplaced expectations from each other, it seems that to solve this issue, interventions and programs other than this approach, i.e., written emotion disclosure, should be implemented.

The results of the studies by Mehrabi et al. (2021) and Mahmoodirad and Bagherian (2015) also showed that occupational stress scores did not decrease in some aspects such as conflicts with nurses and conflicts with doctors, responsibility and physical environment.

Occupational stress, as a whole, has numerous and various dimensions, each requiring attention. Since each of these dimensions alone can be an effective variable on nurses’ lives, it is necessary to make appropriate and effective plans in regard with each. As the literature review yielded different results, it is not possible to say which dimensions depend on which interventions. It seems that the results are different based on the type of intervention, the type of research, the target population, and the time and the place of the study, and everything should be planned according to each specific research or location. In fact, everything should be carried out in an organizational manner; in every organization and every job group, interventions should be developed according to the dimensions with lower scores, in which interventions have been effective.

## Research limitations

One of the limitations of the current research was the outspread of COVID-19 pandemic which both increased nurses’ stress levels and made the sampling process difficult and challenging. Another challenge was the presence of the intervention and the control groups’ subjects in the same environment, which could have led to sample contamination; the control group subjects could probably become aware of the interventions performed in the intervention group.

The intervention was run for 8 weeks because the nurses of the ICU and CCU had very high workload due to a shortage of manpower, then prolonging the duration of the intervention due to current organizational constraints in hospitals, was perceived extra burden on nurses and therefore be considered one of the limitations of this study. Liao and Secemsky (2015) also referred to the local and systems factors and limited opportunities to engage in narrative medical writing among medical residents.

## Study’s strengths

One of the strengths of the study is the selection of two intervention and control groups and the block division of the subjects, and another strength is the pre-test in both intervention and control groups.

## Final conclusion

The results showed that written emotion disclosure has an impact on the intensive care unit nurses’ level of occupational stress and the related dimensions, including death and dying, inadequate emotional preparation, peer-related problems, supervisor-related problems, workload, treatment uncertainty, patients and their families, and discrimination, except for the dimension conflicts with physicians. This means that using this treatment plan can have many benefits as a simple, inexpensive, feasible, and effective non-pharmacological nursing intervention. As the nurses involved in patient care are sometimes ignored, whereas they provide permanent care for critically ill patients, especially in special care units, and constantly observe patients’ pain, suffering, complications, and death, their level of stress increases; it is destructive and harmful to them. On the other hand, the coincidence of such stress in the work place with the stress caused by the COVID-19 pandemic has made the condition intolerable, making nursing interventions more necessary than ever. Therefore, the nurses working in intensive care units are recommended to get familiar with such approaches to contribute to self-help and improve the quality of nursing services in patient care.

The main finding of this research can be provided to the professors, nursing managers and health trustees of medical sciences universities, supervisors and nurses working in special care units and other medical staff of health and treatment centers in order to pay attention to nurses and their psychological problems, use interventions such as rewriting pleasant events to reduce them.

It is suggested that the present study should be conducted in other samples with different demographic characteristics and its effectiveness should be compared with other existing samples.

## Data availability statement

The original contributions presented in the study are included in the article/supplementary material, further inquiries can be directed to the corresponding author.

## Ethics statement

The studies involving human participants were reviewed and approved by ethics committee of Ahvaz Jundishapur University of Medical Sciences. The patients/participants provided their written informed consent to participate in this study.

## Author contributions

All authors listed have made a substantial, direct, and intellectual contribution to the work and approved it for publication.

## Conflict of interest

The authors declare that the research was conducted in the absence of any commercial or financial relationships that could be construed as a potential conflict of interest.

## Publisher’s note

All claims expressed in this article are solely those of the authors and do not necessarily represent those of their affiliated organizations, or those of the publisher, the editors and the reviewers. Any product that may be evaluated in this article, or claim that may be made by its manufacturer, is not guaranteed or endorsed by the publisher.
